# Genetic determinants of hair and eye colours in the Scottish and Danish populations

**DOI:** 10.1186/1471-2156-10-88

**Published:** 2009-12-30

**Authors:** Jonas Mengel-From, Terence H Wong, Niels Morling, Jonathan L Rees, Ian J Jackson

**Affiliations:** 1 Section of Forensic Genetics, Department of Forensic Medicine, Faculty of Health Sciences, University of Copenhagen, Denmark; 2 Department of Dermatology, University of Edinburgh, 1/F, Lauriston Building, Lauriston Place, Edinburgh, EH3 9HA, UK; 3 MRC Human Genetics Unit, Institute of Genetics and Molecular Medicine, Western General Hospital, Edinburgh, EH4 2XU, UK

## Abstract

**Background:**

Eye and hair colour is highly variable in the European population, and is largely genetically determined. Both linkage and association studies have previously been used to identify candidate genes underlying this variation. Many of the genes found were previously known as underlying mutant mouse phenotypes or human genetic disease, but others, previously unsuspected as pigmentation genes, have also been discovered.

**Results:**

We assayed the hair of a population of individuals of Scottish origin using tristimulus colorimetry, in order to produce a quantitative measure of hair colour. Cluster analysis of this data defined two groups, with overlapping borders, which corresponded to visually assessed dark versus red/light hair colour. The Danish population was assigned into categorical hair colour groups. Both cohorts were also assessed for eye colour. DNA from the Scottish group was genotyped at SNPs in 33 candidate genes, using 384 SNPs identified by HapMap as representatives of each gene. Associations found between SNPs and colorimetric hair data and eye colour categories were replicated in a cohort of the Danish population. The Danish population was also genotyped with SNPs in 4 previously described pigmentation genes. We found replicable associations of hair colour with the *KITLG *and *OCA2 *genes. *MC1R *variation correlated, as expected, with the red dimension of colorimetric hair colour in Scots. The Danish analysis excluded those with red hair, and no associations were found with *MC1R *in this group, emphasising that *MC1R *regulates the colour rather than the intensity of pigmentation. A previously unreported association with the *HPS3 *gene was seen in the Scottish population. However, although this replicated in the smaller cohort of the Danish population, no association was seen when the whole study population was analysed.

**Conclusions:**

We have found novel associations with SNPs in known pigmentation genes and colorimetrically assessed hair colour in a Scottish and a Danish population.

## Background

The colours of hair, skin and eyes provide some of the most visible variation between and within human populations. Whilst variation in skin pigmentation is notable between populations, hair colour variation is most notable within populations of European origin [1,2]. In Europeans, genetic factors explain 92% of the variation in hair colour, while most of the rest of the variation is due to environmental influence [2]. Blond and red hair colours are commonly seen variations in Europeans, but are rare in other populations. Both linkage studies in families and genome-wide association studies in populations have identified genetic factors that determine hair and eye colour. Thus, for example, brown eye and hair colour was mapped to chromosome 15 by linkage [3,4] and was also found associated with SNPs in the *OCA2*, and adjacent *HERC2 *genes in both whole genome and candidate studies [4][5][6][7][8]. Red hair colour was initially attributed to *MC1R *variation by association studies [9] but the highly penetrant phenotype of some variants subsequently allowed family studies [10]. Presently, more than 70 variations in *MC1R *have been reported [11]. The commonest variants in *MC1R *have been characterised as highly penetrant or low penetrant red hair alleles and classified as *R *or *r*, respectively [12]. Red-haired individuals most commonly are *R*/*R *genotype, and this genotype accounts for as much as 84% of red hair colour, but *R*/*r*, *R*/+ and *r*/*r *genotypes can also result in red hair [12][13][14]. Association analyses between hair colour and SNPs in Europeans have been more informative than linkage analyses and have revealed associations with several genes, most already known from mouse or human pigmentary mutations; *SLC45A2*, *TYR*, *OCA2*, *KITLG, ASIP*, and *TYRP1 *but others not previously implicated in pigmentation; *SLC24A4, IRF4 *and *TPCN2 *[4,5,15,16][17]. In addition, recently four groups have independently found strong associations between eye colour and polymorphisms in the *HERC2 *gene upstream of *OCA2 *[5][6][7][8]. These SNPs also have a weak association with hair colour, but there are notably stronger associations with hair colour and haplotypes across the *OCA2 *gene itself [4].

The precise mechanisms behind most SNP associations with hair colour are not clear. However, most associations found using whole-genome studies map in or near to genes which are already known to play a role in pigmentation through human, mouse or zebrafish mutations. It is clear that most components of the pigmentation pathway are already known through mutation and disease studies and that subtle changes in expression or function of these genes underlie much of normal pigmentary variation.

On this principle, we have studied associations between 384 tagging SNPs which comprehensively cover 33 candidate genes known to be involved in the pigmentation pathway from mouse and human disease genetics or other studies and hair and eye colour in a Scottish population. Associations with hair colour were followed up in a Danish population using 25 SNPs in regions of four candidate pigmentation genes and hair colour.

## Methods

### The Scottish population

A total of 133 unselected young adults of ages ranging from 18 to 40 (32 males and 101 females) were recruited from Edinburgh, Scotland. Their hair colour was measured from 6 scalp hair sites: left and right frontal (8 cm superiorly from supraorbital ridge), left and right temple (8 cm laterally from supraorbital ridge), left and right occipital (5 cm laterally from occiput) using tristimulus L*a*b* colorimetry with a Minolta spectrophotometer CM-2600d (Minolta Co., Ltd, Osaka, Japan). Means of triplicate measurements over all sites were taken. Colour was represented as summary values in three dimensions designed to be commensurable with human colour perception: L*, representing lightness, on a scale of 0-100 where 0 is black and 100 is white; a*, representing red-green, on a scale from +60 to -60, where positive values indicate increasing shades of red; and b*, representing a yellow-blue, on a scale from +60 to -60, with positive values representing increasing shades of yellow. These three values were plotted and intersected into a three dimensional space to give a numerical value for colour. Subjects with dyed hair and/or who were not of north-European origin were excluded from the study. The eyes of the participants were photographed and categorized into blue, grey, green hazel or brown by inspection. Table 1 shows the numbers of subjects by eye colour, pooled to give sufficient statistical power, with their hair colour by observation. From all volunteers, venous blood was collected and DNA was extracted using Nucleon Genomic DNA extraction kit (Tepnel Life Sciences PLC, Manchester, UK). Ethical approval was obtained from the Lothian Regional Ethics Committee, and consent to carry out and publish the study obtained from each subject.

**Table 1:** Eye and hair colour of 133 Scots in this study

	Black hair	Brown hair	Fair hair	Red hair	Totals
Blue/grey eyes	3	19	21	7	50

Brown/hazel eyes	18	31	2	7	58

Green eyes	2	13	4	6	25

Totals	23	63	27	20	133

### The Danish population

Unrelated, healthy donors participated and their hair colours categorised as white, light blond, dark blond, brown, black, red or auburn by the same observer. Elderly participants answered by recall of their colour at age 20 years. Data and genotypes from 378 participants with hair colour other than red were analysed with respect to dark or blond hair colour and were tested at 25 SNPs. Initial validation of SNP associations with hair colour in the Scottish population used a subset of participants from the Danish cohort (N = 210) with subsequent follow up in the whole sample. The hair colours were classified as dark (dark blond, brown, black) or light (fair, light blond). Table 2 shows the hair and eye colour categories from this population. Participants were asked where their parents or grandparents were born to determine the individual's ancestral origin, and only those of north European ancestry were included. Blood samples were collected from all volunteers and DNA was purified using the QIAamp DNA blood minikit according to the manufacturer protocol (Qiagen). The project was approved by the Danish ethical committee (ref. KF-01-037/03), and consent to carry out and publish the study obtained from each subject.

**Table 2:** Eye and hair colour of 382 Danes in this study

	Black/brown/dark blond hair	Light blond/fair hair	Red/auburn hair	Totals
Blue/grey eyes	111	146	11	268

Brown/hazel eyes	51	14	4	69

Green eyes	28	15	2	45

Totals	190	175	17	382

In this population the sample size of 210 achieves 67% power to detect at P = 0.05 an association with an odds ratio of 1.6 for a SNP with a minor allele frequency of 0.45 (as we see, for example with rs2254913 in *HPS3*)

### Candidate genes and SNP selection

A total of 33 candidate genes were selected which are reported to play a role in pigmentation through melanin biosynthesis, metabolic pathways associated with pigmentation or tanning response or melanocyte biology. The candidate genes were: *ASIP, BLOC1S3, CYP1A2, CYP2C8, CYP4B1, DCT, DTNBP1, ERCC1, ERCC2, ERCC3, ERCC4, ERCC5, ERCC6, ERCC8, GNAS, GPR143, HPS1, HPS3, HPS4, HPS5, HPS6, KIT, KITLG, MITF, MYO5A, OCA2, PRKAR1A, SLC24A5, SLC45A2, SOX10, TP53, TYR *and *TYRP1*. SNPs from these and other genes were screened using data from CEU in HapMap, viewed in Haploview, http://www.hapmap.org. Tagging SNPs were selected as representatives of the candidate gene as an alternative to analyzing all known SNPs in the candidate genes and to minimize the chance of analysis SNPs in strong linkage disequilibrium with each other.

The choice of SNPs was refined by looking at frequency in the European population and suitability for analysis on the Illumina platform. The Illumina Assay Design Tool was used to refine and eliminate cross hybridisation and improve success rate. SNP selection used design score, design rank, minor allele frequency (MAF) and validation status. MAF, Golden Gate validation status and SNP scores were obtained from Illumina.

### SNP typing with Illumina microarray (Scots)

The Illumina GoldenGate microarray system was used for genotyping of the Scottish samples [18]. Typing was performed at the Wellcome Trust Clinical Research Facility, Institute of Genetics and Molecular Medicine, Edinburgh [19].

### SNP typing by MALDI-TOF MS (Danes)

A multiplex PCR with 13 short amplicons was designed to amplify the loci with the selected 13 candidate variations [see Additional file 1, Table S1]. Primer concentrations ranging from 26.7 μM to 66.7 μM [see Additional file 1, Table S2]. The reaction was balanced to obtain equal peak intensities in the MALDI-TOF MS spectra. PCRs and detection by MALDI-TOF MS technology were performed as previously described [14].

### Sequencing of the *MC1R *gene

Sequencing of *MC1R *was performed as previously published [10]. *MC1R *alleles were classified according to the *R *nomenclature for high penetrance 29insA, D84E, R142H, R151C, R142 R160W and D294H. The low penetrance alleles V60L and V92M were classed as *r *[12,20].

### Statistical analysis

Allele frequencies of categorical data were analysed using Fisher's exact test and odds radios. Quantitative values were analysed using linear regression on SNP allelic counts and SNP effects tested by Walds test using Plink v0.99 [21]. All P-values were corrected by using empirical (adaptive) permutation with standard settings or by using Bonferroni single-step adjusted p-values. Analysis of variance (ANOVA), cluster analysis and discriminant analysis were performed with SYSTAT^® ^v.11. Kruskal-Wallis one way analysis of variance was used to test for differences between male and female a* values. Linkage disequilibrium (D' and R^2^) was calculated using Haploview 4.0 [22].

## Results

### Quantitative measure of hair colour

**Figure 1:**
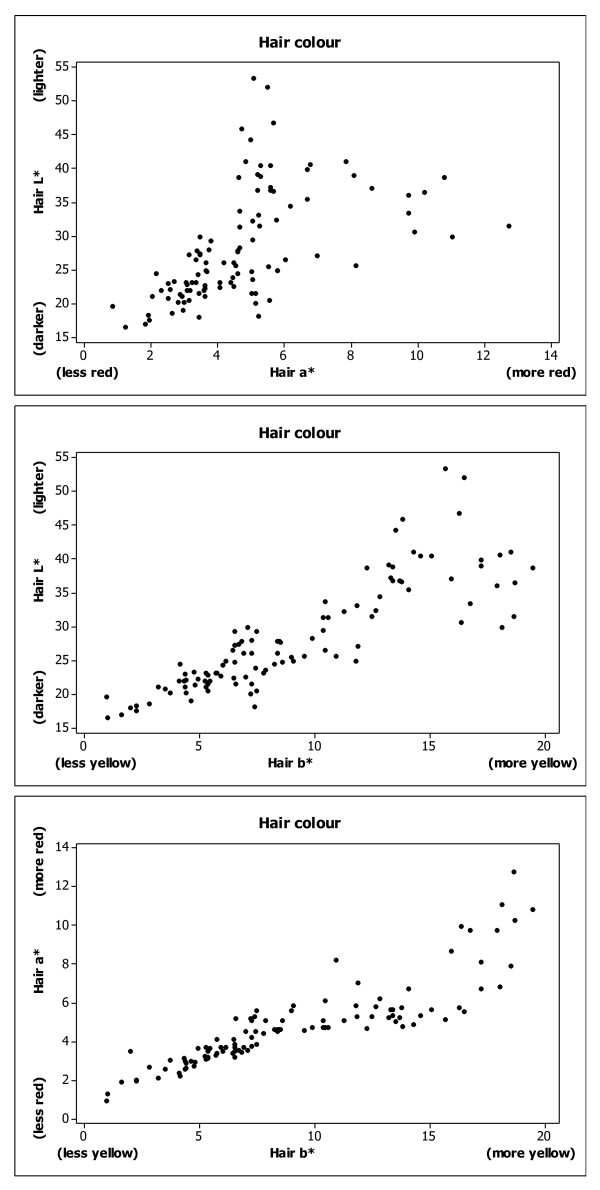
L*(dark/light) a*(red) b*(yellow) values (hair colour) measured by tristimulus colorimetry in 107 Scots from Edinburgh. Each individual is plotted as L* vs a* (top), L* vs b* (middle) and a* vs b* (bottom).

**Figure 2:**
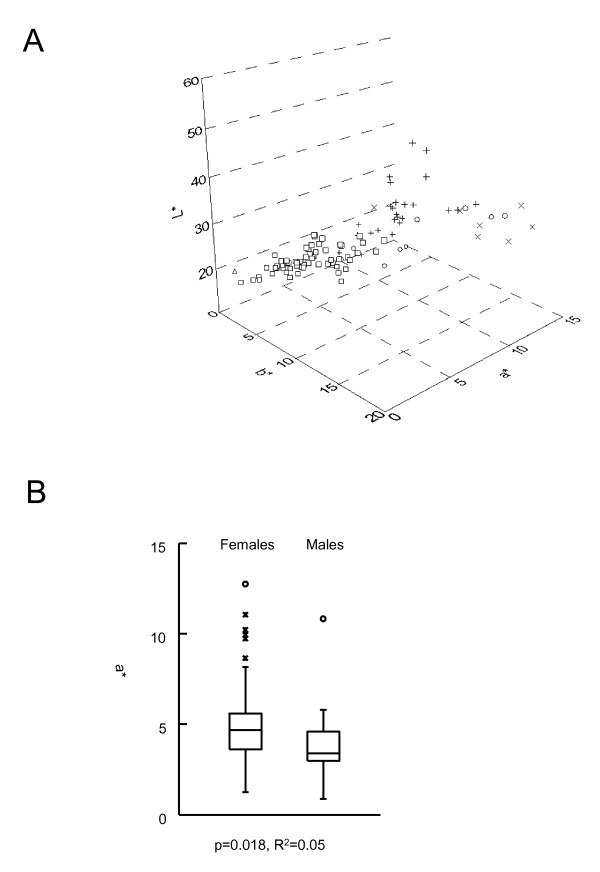
A: L*(dark/light) a*(red) b*(yellow) values (hair colour) plotted in 3D colour space. The observer report of hair colours were categorised as black (△), brown (€), blonde (+), red (X) and auburn (O). B: Box plot of a* distribution among males and females. Significant deviation of the means was calculated using ANOVA.

We measured hair colour in the Scottish population using tristimulus colorimetry, which assigns three values; L* assays the light/dark axis, a* measures red/green and b* indicates yellow/blue. Figure 1 shows all individuals plotted against each pair of parameters (L* vs a*, L* vs b* and a* vs b*), The values are clearly correlated, as previously reported [23]. The measured hair colours showed L* values ranging from 16.42 to 53.20 (within a maximum range of 0 to 100, where higher values are lighter). The a* values ranged from 0.88 to 12.74 and b* values from 0.97 to 19.51 (where positive values indicate increasing red and yellow colour respectively). When viewed plotted in 3 dimensions, against each parameter simultaneously, (Figure 2A) the individual hair colours are distributed in a triangular pattern where individuals with brown hair colour had low L*, a* and b* values and red haired individuals had high a* and b* values and midrange L* values. Individuals with blond hair colour had midrange a* and high b* and L* values. A similar hair colour distribution was previously described [23]. Cluster analysis in two groups of the quantitative measures of hair colour defined a group with dark hair colour with 95% confidence intervals L*(16.90;29.80), a* (1.29;6.31) and b* (1.51;10.99) which was separated from the red/light hair colour group L*(26.61,48.61), a* (2.31;11.01) and b* (9.72;19.87), but the groups had merging borderlines at all three dimensions L*(26.61-29.80)/a*(2.31-6.31)/b*(9.72-10.99). Cluster analysis with more than two groups did not correlate with groups as defined by visual inspection. A gender deviation was observed. In females (N = 85), the a* values were significantly higher than for males (N = 23) p = 0.018, R^2 ^= 0.05 (Figure 2B). The mean female a* value is 4.67 and mean male is 3.39. Using a nonparametric test to test for differences, Kruskal-Wallis chi-squared analysis gives a P value of 0.003.

Discriminant analysis showed that the hair colours assigned as blond by inspection were correctly assigned in 85% (N = 22) of the individuals, whereas dark (black, brown) was correctly assigned in 94% (N = 61). Red hair colour was correctly assigned in 86% (N = 6) of the individuals whereas auburn hair colour was classified correctly in only 22% (N = 2) and alternatively defined as either blond (N = 1), brown (N = 3) or red (N = 3).

Chi-square analysis of the hair and eye colour categories (Table 1 and 2) indicates that these are not independent and blue/grey eyes are more often found with fair or blond hair and brown/hazel eyes with dark hair. (Scots, Χ^2 ^= 34.41, 9 d.o.f., P < 0.001; Danes, Χ^2 ^= 29.33, 6 d.o.f., P < 0.001)

### SNP Associations with Hair and Eye Colour

107 individuals from the Scottish cohort were typed at 384 SNPs from 33 genes. Choice of SNPs was guided by HapMap so that each gene was represented by tagging SNPs. In addition the *MC1R *gene was sequenced from each individual and any variants found categorised as *R *(high penetrant red hair) or *r *(low penetrant red hair). Associations between SNPs and quantitative tristimulus values were examined using linear regression and p-values for putative associations were corrected using permutation or by Bonferroni adjustment. Table 3 shows SNPs in 11 genes in which SNPs were found with adjusted p-values of <0.05. Associations of the same 384 SNPs with eye colour were also analysed in the same 107 Scottish individuals, using Fisher's exact test on the categorical data (Table 4). All association data is tabulated in additional material [see Additional file 2]. The Danish population was initially typed at SNPs in 4 genes previously indicated to have associations with hair colour (*SLC45A2*, *HERC2*, *OCA2 *and *MC1R*) across 378 individuals, and associations tested with dark versus light hair (Table 5). A subset of 210 Danes was further analysed to follow up significant associations found in the Scottish population (Table 6). The size of these sample populations mean that for alleles of low frequency the power to detect associations is limited, unless the effect is large.

**Table 3:** Associations between SNP alleles with minor allele frequencies and quantitative measures of the hair colours (L*a*b* values) with P < 0.05 in 107 Scots from Edinburgh.

Gene	Chr.	Position^a^	SNP	Min. allele	MAF^b^	L* p-value	L* R^2^	a* p-value	a* R^2^	b* p-value	b* R^2^	Hair colour
*HPS3*	3	Int 1.1	rs4681169	A	0.30			0.046	0.039			Dark
		Int 1.2	rs16861514	T	0.15			0.043	0.039			Dark
		Int 6	rs16861552	C	0.33			0.021	0.054			Dark
		Int 8	rs6785780	T	0.41			0.003	0.079			Red/Light
		Int 13	rs7636389	A	0.07			0.026	0.049			Red/Light

*DTNBP1*	6	Exon 10 (S272P)	rs17470454	A	0.05	0.011	0.060			0.011	0.044	Light

*TYRP1*	9	Int 6	rs17346161	T	0.06			0.046	0.037			Red/Light

*ERCC6*	10	Int 5	rs1018603	C	0.11	0.041	0.041			0.041	0.038	Dark
		
		Int 9	rs4253162	A	0.07	0.011	0.058			0.011	0.060	Dark

*KITLG*	12	Int 1.1	rs1492354	A	0.07			0.0009^d^	0.119			Red/Light
		Int 1.2	rs1907702	G	0.20			0.018	0.05			Red/Light
		Int 1.3	rs10777129	A	0.08			0.007	0.06	0.043	0.080	Red/Light
		
*OCA2*	15	Int 1.1	rs7495174	G	0.05	0.02	0.051			0.024	0.040	Dark
		Int 1.2	rs7174027	A	0.06	0.005	0.075			0.005	0.060	Dark
		
		Int 2.1	rs12442147	C	0.11	0.03	0.041	0.010	0.066	0.03	0.065	Dark
		Int 2.2	rs12324648	A	0.07			0.019	0.057			Dark
		Int 2.3	rs1470608	A	0.10			0.012	0.063			Dark
		
		Int 4	rs3794604	A	0.08			0.031	0.042			Dark
		
		Int 5	rs749846	A	0.11			0.025	0.054			Dark
		
		Int 19	rs1375166	A	0.20			0.046	0.039			Dark
		
		Int 23.1	rs2311470	C	0.50			0.005	0.073			Dark
		Int 23.2	rs11858340	A	0.44			0.016	0.053			Red/Light
		Int 23.3	rs6497235	A	0.50	0.03	0.050	0.006	0.069	0.03	0.063	Light
		Int 23.4	rs11074306	A	0.41			0.024	0.048			Dark
		Int 23.5	rs6497233	T	0.41	0.02	0.053	0.0004^e^	0.116	0.021	0.098	Light
		Int 23.6	rs17674017	G	0.42			0.005	0.073			Red/Light
		Int 23.7	rs1498509	C	0.49			0.010	0.060			Dark
		Int 23.8	rs11631195	A	0.37	0.004	0.079	0.0002^d^	0.126	0.0037^c^	0.130	Light
		Int 23.9	rs3947367	G	0.29	0.012	0.059			0.012	0.039	Light
		Int 23.10	rs11637518	G	0.35	0.014	0.051	0.009	0.064	0.014	0.078	Light
		Int 23.11	rs989869	C	0.38	0.008	0.063			0.0077	0.053	Dark
		Int 23.12	rs1603784	A	0.23	0.007	0.063			0.0069	0.041	Dark
		
*MYO05*	15	Int 1.1	rs1615028	C	0.17	0.046	0.037					Light
		Int 1.2	rs1724593	G	0.15	0.012	0.059			0.012	0.066	Light
		Int 1.3	rs7176061	C	0.48	0.046	0.041					Dark
		
		Int 2	rs1724625	T	0.30	0.036	0.044					Light
		
		Int 5	rs1724630	G	0.21	0.049	0.039			0.049	0.040	Light
		
		Int 9	rs1632403	T	0.08	0.042	0.039			0.042	0.049	Light

*MC1R*	16	Exon	R	None	0.21			2.00 × 10^-6c^	0.317	0.047^c^	0.156	Red
		Exon	r	None	0.23			0.005	0.07			Dark/Light

*PRKAR1A*	17	Int 2	rs2952275	T	0.36	0.047	0.036					Light
		
		5' UTR	rs8080306	C	0.27	0.021	0.047					Light

*GNAS*	20	Int 3	rs2295583	T	0.28	0.03	0.43					Light
		
		Int 5	rs3730168	A	0.41	0.015	0.052					Dark
		
		Int 6	rs919197	T	0.43	0.006	0.07			0.0062	0.059	Light

*HPS4*	22	Int 2	rs9613187	T	0.11	0.012	0.059			0.012	0.037	Light
		
		Int 5	rs17401652	T	0.10			0.028	0.047			Red/Light

a: Intron (Int), Exon or Untranslated region (UTR) position in the gene

b: Minor allele frequency (MAF)

c: Significant using Bonferroni correction. Other L*, a* and b* values were corrected using empirical permutations

d: Significant using False Discovery Rate correction

**Table 4:** Associations between SNP alleles with minor allele frequencies and eye colour categorised from photos of 107 Scots from Edinburgh.

Gene	Chr	SNP	**position**^a^	Min A	**MAF**^b^	Ranked Eye colours	Shades		Brown vs blue		Brown vs other	
						
						p-values	p-values	OR	p-values	OR	p-values	OR
*HPS3*	3	rs7643410	int 1	G	0.08						0.04	2.9
		rs2254913	int 5.1	A	0.37	0.006	0.03	1.7	0.003	2.6	0.01	1.9
		rs2689229	int 5.2	A	0.46	0.01	0.02	1.8	0.004	2.5		
		rs2689230	int 5.3	G	0.23	0.04	0.046	2.2	0.02	2.6	0.04	2.1
		rs2689234	int 6	G	0.49	0.03			0.009	2.3		
		rs6785780	int 8	T	0.41	0.03			0.02	2.3		
		rs2681092	int 15	T	0.38	0.02			0.01	2.3	0.04	0.5

*KIT*	4	rs17084733	3' UTR	A	0.11				0.02	4.3	0.04	3.6

*DTNBP1*	6	rs9476886	int 1	T	0.28				0.04	2.4		

*ERCC8*	5	rs4647128	int 10	G	0.03	0.04	0.02	6.1				
		
		rs4235483	int 9	A	0.44		0.04	1.7				

*ERCC6*	10	rs4253231	3'UTR	C	0.09	0.007	0.003	3.6	0.08	2.8	0.01	3.0

*CYP2C8*	10	rs11572177	int 8	G	0.30						0.04	1.9

*DCT*	13	rs9584233	int 6	T	0.11				0.03	3.4	0.03	2.4

*OCA2*	15	rs7495174	int 1.1	G	0.05	0.001^d^	0.006	6.3	0.003	NA	0.009	5.9
		rs7174027	int 1.2	A	0.06	0.009		3.1	0.003	NA	0.03	3.3
		
		rs7179994	int 2.1	G	0.14	0.03			0.03	3.1	0.04	2.3
		rs1597196	int 2.2	T	0.15	0.008	0.03	2.0	0.008	3.6	0.02	2.4
		rs1470608	int 2.3	A	0.10						0.046	2.3
		rs12324648	int 2.4	A	0.07	0.006	0.04	2.9	0.009	5.2	0.004	4.1
		
		rs3794604	int 4	A	0.08	0.03		2.5	0.04	3.8	0.006	3.7
		
		rs746861	int 6	C	0.45		0.02	1.8				
		
		rs7176632	int 16	T	0.18	0.0003	0.0008	2.9	0.0008	5.0	0.01	2.3
		
		rs7173419	int 18.1	T	0.27	0.01	0.02	2.1	0.046	2.2		
		rs2594938	int 18.2	C	0.26	0.01	0.02	2.0	0.02	2.3		
		rs1562592	int 18.3	T	0.17	5.21 × 10^-5d^	4.12 × 10^-5d^	3.7	0.0009	4.2	0.0009	2.9
		rs1448490	int 18.4	A	0.16	0.03	0.02	2.8	0.009	7.0	0.002	8.6
		
		rs17674017	int 23.1	G	0.42	0.008	0.04	1.9	0.03	2.2		
		rs1498509	int 23.2	C	0.49	0.03			0.04	2.0		
		rs11631195	int 23.3	A	0.37	0.04			0.04	2.0		
		rs989869	int 23.4	C	0.38	0.02						
		rs1603784	int 23.5	A	0.23	0.01			0.03	2.6		
		rs11074304^c^	int 23.6	A	0.42	0.04			0.04	2.2		

*GNAS*	20	rs4810147	int 1	A	0.49	0.02			0.004	2.6	0.04	1.8

a: Intron (Int), Exon or Untranslated region (UTR) position in the gene

b: Minor allele frequency (MAF)

c: alleles slightly out of Hardy Weinberg Equilibrium

d: Significant using Bonferroni correction. Other p-values were corrected by empirical permutation

e: Significant association in Duffy et al. 2006

NA: No answer. Only observed among individuals with brown eye colour

**Table 5:** Associations between SNP alleles and dark versus light hair colour in 378 Danes.

Gene	Chr.	Position^a^	SNP	**Min.allele**	MAF^b^	p-value	OR^e^	Hair colour
*MATP*	5	Exon 5 L374F	rs16891982	C	0.02	0.005	7.0	Dark
		Exon 3 K272E	rs26722	A	0.02	0.07		Dark

*HERC2*	15	Int 12	rs916977^c^	T	0.07	8.3 × 19^-5 d^	3.7	Dark
		3'UTR	rs1129038^c^	C	0.11	2.0 × 10^-6 d^	3.5	Dark
		Exon 78 Q3989Q	rs11636232	T	0.46	0.098		
		Int 44	rs2238289	G	0.07	2.2 × 10^-5 d^	4.0	Dark
		Int 56	rs7170852	A	0.09	1.7 × 10^-5 d^	3.5	Dark

*OCA2*	15	Exon 9 W305R	rs1800401	T	0.04	0.75		
		Exon 13 Q419R	rs1800407	A	0.04	0.31		

*MC1R*	16	5' UTR	rs3212359	A	0.34	0.17		
		5' UTR	rs3212361	T	0.27	0.055		
		V60L	rs1805005	A	0.12	0.20		
		V92M	rs2228479	A	0.07	0.22		
		R151C	rs1805007	T	0.09	0.70		
		R160W	rs1805008	T	0.08	0.055		
		R163Q	rs885479	T	0.04	1.0		
		T314T	rs2228478^c^	G	0.09	1.0		

a: Intron (Int), Exon or Untranslated region (UTR) position in the gene

b: Minor allele frequency (MAF)

c: alleles slightly out of Hardy Weinberg Equilibrium

d: Significant using Bonferroni correction. Other p-values were corrected by empirical permutation

e: Odds ratios are only shown for statistically significant associations

**Table 6:** Associations between SNP alleles and dark versus light hair colour in 210 Danes.

Gene	Chr.	**Position**^a^	SNP	Min. allele	**MAF**^b^	Fisher's Exact	**OR**^d^	Hair colour
*HPS3*	3	Int 5	rs2254913^c^	A	0.45	1.00		
		
		Int 8	rs6785780	T	0.44	0.048	1.6	Light

*DTNBP1*	6	Exon 10 S191P	rs17470454	A	0.07	0.32		
		
		Int 6	rs6909929	A	0.42	0.37		

*ERCC6*	10	3'UTR	rs4253231	C	0.10	0.56		

*CYP2C8*	10	Int 8	rs11572177	G	0.27	0.60		

*TYR*	11	Int 3	rs12421746	T	0.04	0.043	3.1	Light

*KITLG*	12	Int 1	rs10777129	A	0.07	0.013	3.0	Light
		Int 1	rs1492354	A	0.08	0.031	2.5	Light

*DCT*	13	Int 6	rs9584233	T	0.12	1.00		

*GNAS*	20	Int 1	rs4810147^c^	A	0.50	0.60		
		
		Int 6	rs234630	C	0.23	0.38		
		Int 6	rs919197	T	0.50	0.86		

a: Intron (Int), Exon or Untranslated region (UTR) position in the gene

b: Minor allele frequency (MAF)

c: alleles slightly out of Hardy Weinberg Equilibrium

d: Odds ratios (OR) are only calculated for statistically significant associations

### The *KITLG *gene and hair colour

**Figure 3:**
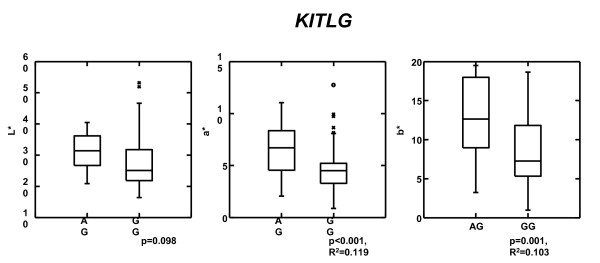
Box and whiskers plot of tristimulus colorimetry L*, a* and b* values (hair colour) of Scottish individuals (N = 107) with the *KITLG *SNP rs1492354. Significance and variance was calculated using ANOVA.

In the Scottish population, the three SNPs, rs1492354 (p = 0.0009), rs1907702 (p = 0.018) and rs10777129 (p = 0.007) located in intron 1 of the *KITLG *gene were significantly associated with hair colour (Table 3). Of the three SNPs, the rs1492354 AG genotype showed the highest contribution to both the red hair colour dimension a* values (P < 0.001, R^2 ^= 0.119) and the yellow hair colour dimension b* values (p = 0.001, R^2 ^= 0.103), the latter value was significant when uncorrected by permutations. The L* axis also showed a trend towards lighter hair colour associated with the A genotype, although this is not statistically significant (Figure 3). Associations were also found between hair colour (a* values) and the two other intron 1 SNPs, rs1907702 and rs10777129. All three SNPs are in linkage disequilibrium in the population, with D' and r^2 ^values as follows: rs1492354 and rs1907702 (D' = 1.0, r^2 ^= 0.33), rs1492354 and rs10777129 (D' = 0.88, r^2 ^= 0.74), rs1907702 and rs10777129 (D' = 1.0, r^2 ^= 0.34)

When retyped in the Danish population, the SNPs rs10777129 (p = 0.02, OR = 3.0) and rs1492354 A (p = 0.04, OR = 2.5) were significantly associated with light hair colour (Table 4). In both the Scottish and the Danish populations, the alleles associated with lighter colour had similar low frequency (rs1492354: 0.08 and rs10777129: 0.07), and given the sample sizes only one homozygote rs10777129 GG was observed, in a Dane.

### The *OCA2 *gene and eye and hair colour

SNPs in or close to the *OCA2 *gene have been reported to be associated with hair and eye colour. However, reported associations are with different regions of the gene; including coding variations and SNPs within the adjacent *HERC2*. In the Scottish population, associations between eye colour and tagging SNPs were found for SNPs in introns 1, 2, 4, 6, 16, 18 and 23 of *OCA2 *(Table 4). Associations with hair colour were also found for some of these same SNPs, and others, located in introns 1, 2, 4, 5, 19 and 23 (Table 3). The intron 1 SNPs rs7495174 (p = 0.02, R^2 ^= 0.051) and rs7174027 (p = 0.005, R^2 ^= 0.075) were associated with the L* values, whereas SNPs in intron 2, 4, 5 and 19 were associated exclusively or more strongly with the a* values. The majority of SNPs associated with hair colour were located in intron 23 and SNPs in this intron were associated with both a* and L* values. The markers with the highest correlation were found in intron 23 for rs6497233 (p = 0.0004, R^2 ^= 0.116) and rs11631195 (p = 0.0002, R^2 ^= 0.126) (Table 3). Subsequently, analysis of variance of the rs11631195 AA versus the AG and GG collapsed genotypes showed significant association on the black/white dimension L* value (p = 0.001, R^2 ^= 0.094), the red dimension, a* value (p = 0.009, R^2 ^= 0.064) and the yellow dimension, b* value (p = 0.001, R^2 ^= 0.107). LD analysis of the data reveals that in this population the SNPs in introns 1 to 5 are in linkage disequlibrium, as are those in introns 19 to 23 (data not shown). Thus there appears to be two separate regions of association with hair and eye colour in the OCA2 gene.

As we and others have previously published, a SNP in a putative regulatory element for *OCA2*, located about 20 kb upstream within the *HERC2 *gene, is associated with eye colour [4][5][6,7] and hair colour [4]. In the Danish population (N = 378) analysed here, a significant association was also observed between dark hair colour and SNPs in *HERC2 *(rs916977, rs1129038, rs2238289 and rs7170852) (p = 2.0 × 10^-6 ^- 8.3 × 10^-5^, OR = 3.5-4.0). However, the *OCA2 *coding SNPs R419Q (rs1800407) and R305W (rs1800401) were not significantly associated with hair colour (Tables 5), although the minor allele frequencies were low (0.04 for each) which limits the power to detect associations in populations of this size.

### The *MC1R *gene and hair colour

Sequence variations in *MC1R *in the Scottish population were categorized into the two allelic groups *R *and *r*. The *R *group was significantly associated (p = 2.0 × 10^-6^, single step adjusted Bonferroni corrections) with high correlation with a*-values (R^2 ^= 0.317) that are characteristic of red hair colour. The *MC1R *'*r*' group was significantly associated with low a*-values (p = 0.005, R^2 ^= 0.007) when *R *and consensus sequences were collapsed. Analysis of variance of the *MC1R R*/*R *genotype showed significant association for both the red dimension, a* value (P < 0.001, R^2 ^= 0.367) and the yellow dimension, b* value (P < 0.001, R^2 ^= 0.163). No association was seen with the L*, light-dark, dimension. In addition, the Danish population excluded individuals with red hair, which permitted an analysis of association between individual *MC1R *variants and light hair. No association was seen, reinforcing the notion that *MC1R *affects the colour but not the intensity of hair pigmentation.

### The *HPS3 *gene and hair colour

We initially identified in the Scottish population a hitherto unreported association between the *HPS3 *gene and the red/yellow colour axes. Five SNPs (rs4681169, rs16861514, rs16861552, rs6785780 and rs7636389), all in LD in this population, were significantly associated with the red dimension a* value of which rs6785780 (p = 0.003, R^2 ^= 0.079) was the most strongly associated (Table 3). When replicated initially in a subset (N = 210) of the Danish population, the association between the rs6785780 T allele and light hair colour was also significant (p = 0.04, OR = 1.6) (Table 4). However, an extended analysis of this SNP on the larger (N = 378) Danish population failed to find an association. Our data do not, therefore, support a role for *HPS3 *in hair colour variation, but suggest that it may be worth further investigation.

### The *TYR *and *SLC45A2 *genes and hair colour

SNPs within the tyrosinase (*TYR*) and the *SLC45A2 *(*MATP*)genes have previously been shown to associate with hair colour [15,16]. We therefore paid particular attention to these genes in our populations. The SNP rs12421746 in the *TYR *gene was significantly associated with blond hair colour in the Danish population (p = 0.04, OR = 3.1) (Table 6), but we did not replicate this association with any of the colorimetric values in the Scottish population. In the Scottish population, the minor allele frequency in *TYR *was 0.012 while it was 0.04 in the Danish population. Again this low allele frequency will restrict the ability to detect small effects in a population sample of the size studied here.

Likewise, no statistically significant association was observed between tagging SNPs in *SLC45A2 *and hair colour in the Scottish population, whereas in the Danish population (N = 378), the one coding SNP rs16891982 (F374L) (p = 0.005, OR = 7.0) was associated with dark hair colour (Table 5).

## Discussion

We have used quantitative hair colour on a tristimulus L*, a* and b* scale not only to associate genetic markers to hair colour, but also to evaluate the accuracy of the inspected hair colours. The two clusters of light or dark hair colour based on colorimetric analysis were well separated and good correlation was observed between inspections (88-94%) and quantitative hair measures. Further sub-classification of these groups did not satisfactorily correlate with the groupings. Shekar and co-workers observed 97% correct classification in two groups whereas only 73.1% were correctly assigned using observer reported colour in six groups [2]

In total, robust associations between hair colour and five genes *MC1R, KITLG, TYR, OCA2 *and *SLC45A2 *were observed. SNPs in or close to all of these have previously been reported by others as showing associations.

MC1R is a melanocyte-specific G-protein coupled receptor for alpha-melanocyte stimulating hormone and is well established as the major determinant of red hair colour [9][10][11][12][13]. Among the numerous variants, those classified as "*R*" are highly penetrant red-hair alleles. In the Scottish population the *MC1R R*/*R *genotype showed the strongest contribution to the variance of the red/yellow correlated dimensions a*(r^2 ^= 0.367)/b*(r^2 ^= 0.163) (Table 3).

Associations with the a* dimensions were also observed with SNPs in intron 1 of the *KITLG *gene. In both the Scottish and Danish populations. Another SNP near to the *KITLG *gene , rs12821256, has also been shown to be associated with hair colour in European populations from Iceland, Netherlands and The United States [4,15]. This SNP is located several hundreds of kilobases 5' of the *KITLG *gene and does not appear to be in LD with those we have analysed in intron 1 (D' = 1, r^2 ^= 0.01). Unfortunately this SNP was not typed in our populations and so we are unable to determine whether those we analysed show a better correlation with hair colour than this previously reported one. *KITLG *encodes stem cell factor, the ligand of the KIT receptor which is essential for normal melanocyte proliferation and development [24]. Mutations of *KITLG *in mice result in deficits in melanocytes and unpigmented patches in the skin and hair and it is not unreasonable to expect that variation in expression or function of the gene in humans could result in variation of melanocyte number in the hair follicles.

The *OCA2 *gene was first identified in mice, in which mutations of the gene result in a pale coat, and was later shown to be identified in patients with tyrosinase-positive albinism. The function of the gene product is not unequivocally established, but it is related to a transporter protein family which has 12 transmembrane domains and is localised to the melanosome [25]. Associations of *OCA2 *and *HERC2 *SNPs with hair and eye colour were found in this study, in accordance with previous reports of linkage or association [3],[4],[5],[6],[7]. The major contribution to eye colour was conveyed by two *HERC2 *SNPs, rs1129038 and rs129138332, which lie about 20 kb upstream of the *OCA2 *gene, and which were almost in perfect linkage disequilibrium [5,6]. Association was observed between hair colour and rs1129038 in the Danish population (p = 2.0 × 10^-6^, OR = 3.5), but two SNPs rs2238289 and rs916977 that were in completely linkage disequilibrium with rs1129038 (D' = 1, R^2 ^= 0.62 and D' = 1, R^2 ^= 0.64) were slightly more strongly associated with dark hair colour (p = 8.3 × 10^-5^, OR = 3.7 and p = 2.2 × 10^-5^, OR = 4.0). These results support earlier results from Shekar and co-workers who demonstrated *OCA2 *haplotypes to be more strongly associated with hair colour than rs1129038 and rs129138332 [3]. By contrast the strongest association with hair colour in the Scottish population in the *OCA2 *gene were accounted for by two SNPs in intron 23 on the red dimension (a*) rs6497233 (p = 0.0004, R^2 ^= 0.116) and rs11631195 (p = 0.0002, R^2 ^= 0.126). Linkage disequilibrium was observed between these markers (D' = 0.94, R^2 ^= 0.79). The association results from the *HERC2*/*OCA2 *region suggest that the molecular mechanism affecting eye colour may not be the same as for hair colour.

Overall in the Danish population, the strongest association with hair colour was observed with the *SLC45A2 *missense variant rs16891982 (OR = 7.0) followed by the *HERC2 *(upstream *OCA2*) variant rs2238289 (OR = 4.0) and the *KITLG *variant rs1492354 (OR = 3.0). Associations between hair colour and SNPs in *TYR *and *SLC45A2 *were only observed in the Danish population. Either of these genes, when mutant in mice or humans, result in albinism. *TYR *is the rate-limiting enzyme required for melanin synthesis whilst *SLC45A2 *is a solute transporter whose substrate is unknown [25]. Absence of observed association with these two genes in the Scottish population may well be due to the limited population sizes. The Danish population has power of 82% to detect at p = 0.05 an effect of this *TYR *SNP with the observed odds ratio of 3.1 and minor allele frequency of 0.04.

We tentatively suggest a novel association between hair and eye colour and the *HPS3 *gene. 7 SNPs across the whole gene showed significant association in the Scottish population. However, although initially replicated in a subset of the Danish population when extended to the whole sample we no longer detect association. *HPS3 *belongs to a group of genes that are involved in biogenesis and/or maturation of multiple cellular organelles including lysosomes and melanosomes, the site of melanin synthesis and export. In the mouse, at least seventeen genes have been identified as HPS like genes, with a mutant phenotype comprising reduced pigmentation and a long bleeding time [26]. Seven of these genes have been demonstrated to be mutated in forms of the inherited, human disorder Hermansky-Pudlack syndrome (HPS), which is a syndrome with multiple disorders including oculocutaneous albinism, bleeding tendency and lysosomal dysfunction [27]. Patients with HPS type 3, and mutant in *HPS*, usually have mild symptoms and mice mutated in the orthologous gene (*Hps3*) have the cocoa phenotype which produces a lighter coat colour and prolonged bleeding time but does not have a lysosomal disorder [28][29][30]. We suggest that variation within the human orthologues of the 17 mouse HPS-like genes merits further detailed analysis as candidates for contributing to hair colour variation.

## Conclusions

We have found novel associations between SNPs in pigmentation genes previously shown to play a role in human hair and eye colour and colorimetrically assessed hair colour in a Scottish and a Danish population.

## Competing interests

The authors declare that they have no competing interests.

## Authors' contributions

All authors have read and approved the final manuscript. JM-F designed and performed experiments, analysed data and wrote the paper, nd TW designed and performed experiments, collected clinical samples and data and analysed data. NM designed experiments and analysed data, JLR designed experiments and analysed data and IJJ designed experiments, analysed data and wrote the paper.
